# Blindfolded adults use mental transformation strategies for spatial scaling of tactile maps

**DOI:** 10.1038/s41598-022-10401-x

**Published:** 2022-04-15

**Authors:** Magdalena Szubielska, Wenke Möhring

**Affiliations:** 1 Institute of Psychology, The John Paul II Catholic University of Lublin, Al. Racławickie 14, 20-950 Lublin, Poland; 2 Department of Educational and Health Psychology, University of Education Schwäbisch Gmünd, Oberbettringer Strasse 200, 73525 Schwäbisch Gmünd, Germany

**Keywords:** Psychology, Human behaviour

## Abstract

The current study tested strategies of spatial scaling in the haptic domain. Blindfolded adults (*N* = 31, aged 20–24 years) were presented with an embossed graphic including a target and asked to encode a target location on this map, imagine this map at a given scale, and to localize a target at the same spot on an empty referent space. Maps varied in three different sizes whereas the referent space had a constant size, resulting in three different scaling factors (1:1, 1:2, 1:4). Participants’ response times and localization errors were measured. Analyses indicated that both response times and errors increased with higher scaling factors, suggesting the usage of mental transformation stratergies for spatial scaling. Overall, the present study provides a suitable, novel methodology to assess spatial scaling in the haptic domain.

## Introduction

### Investigating spatial scaling ability

Spatial scaling constitutes an integral component of navigation tasks and map reading and is defined as "the ability to transform distance information from one representation to another one of a different size"^1^ (p. 271). The majority of previous research on spatial scaling investigated this ability in the visual domain^1–7^. However, considering that maps can similarly be encoded using the haptic sense (as done by blind people), recent studies began to investigate spatial scaling in the haptic domain^8–10^. A typical procedure in studies investigating scaling was that participants were presented with a simple map showing a target and an empty referent space. Then, they were asked to use the information provided in the map in order to locate another target in the referent space. Importantly, sizes between the maps and the referent space varied systematically, with the goal to create different scaling factors and ultimately, to investigate participants’ ability to scale distance information. Using comparable tasks, it has been repeatedly shown that spatial scaling is associated with competencies in STEM-related fields (science, technology, engineering, and mathematics)^1,4,11^, suggesting the importance of this specific skill above and beyond spatial tasks.

### Spatial scaling strategies

Considering the significance of spatial scaling, it is crucial to increase our knowledge about the underlying processes and strategies in order to successfully solve these tasks. Research on spatial scaling has identified that individuals may apply different spatial scaling strategies^12,13^. Using a highly error-prone "absolute" spatial scaling strategy, individuals may encode the target location provided in a map in an absolute way. Regardless of differences in scale, they may match the identical information onto a referent space, which results in a linear increase of errors with increasing scaling factors whereas participants’ response times may remain constant across different scaling factors. A second "relative distance" strategy involves a proportional encoding of spatial information. Individuals who use this strategy may encode relative distances of the target and surrounding objects such as the borders when perceiving a map. Then, they may map an identical relative distance onto the referent space^3,14^, which results in constant errors and response times across different scaling factors. A third "mental transformation" strategy refers to the usage of mental zooming in visual imagery. Similar mental transformation processes were shown in studies investigating mental imagery such as rotation^15^, scanning^16^, or comparing patterns differing in size^17,18^. Individuals who use this strategy may encode the map as a holistic image and then mentally transform the size of the image (zooming it up or down) when performing the spatial scaling task. Analogous to mental imagery literature, mental transformation strategies in spatial scaling tasks may elicit a linear increase in errors and response times with higher scaling factors.

When investigating different scaling strategies and reflecting about their effects on the participants’ response patterns, it becomes clear that several methodological constraints need to be addressed in order to disentangle these strategies. One crucial precondition refers to systematically varying scaling factors. Another precondition refers to assessing participants’ errors as well as response times given that strategies are associated with a differential pattern of these dependent variables. To date, only few studies have met these methodological requirements^5,12,13^. The majority of previous research has typically measured accuracy but not response times^1,2,4,6,9,10^. Other studies have only tested a single scaling factor in a within-subject design^6^, making it difficult to study systematic changes in participants‘ performance as a function of scaling factor. Importantly, when referring to the few studies that did meet these constraints, it seems that adults use mental transformation strategies for spatial scaling^5,13^—at least in the visual domain.

### Research on spatial scaling strategies in the haptic domain: further methodological constraints

In addition to these general methodological requirements when investigating spatial scaling strategies, assessing spatial scaling in the haptic domain involves additional challenges. Several studies have already examined how changing an object size in the haptic domain affects participants’ accuracy^9,10,19–23^. However, these studies do not allow conclusions with respect to the spatial scaling strategies used. In line with research in the visual domain, these studies have often not systematically varied scaling factors or did not measure errors and response times.

Up to now, there is one study that met these constraints in the haptic domain, conducted from Szubielska and Möhring^8^. In this respective study, adults were presented with the map and the referent space simultaneously and asked to encode the target location in the map before indicating the same position in the referent space. This was a typical approach in previous scaling studies in the visual domain^1,4,5^ but showed some disadvantages in the haptic domain. As haptic perception is a sequential process^24,25^, exploring the map by touch took longer for larger maps as compared to smaller maps. Consequently, participants’ exploration times interfered with the time used for scaling spatial information, making it difficult to rely on response times as an indicator for scaling per se (for a detailed discussion, see^8^). Therefore, building on the limitations of this previous research, it seems crucial to separate the exploration process of the map from localizing the target in the referent space. In the present study, we adressed this issue and used a novel three-step approach (for related procedures, cf.^2,6,23^). More concrete, the experimental task consisted of three subsequent stages: learning the map, imagining the map at a given scale (with an assessment of response times at this stage), and giving a response in an empty referent space (with an assessment of errors at this stage). Using this novel approach, we aimed to assess scaling strategies in the haptic domain.

Based on evidence suggesting abstract (spatial) representations across the auditory and haptic modality in the human brain^26^ as well as for the visual and haptic modality^27^, it seems reasonable that participants may apply mental transformation strategies in the haptic domain. This expectation is supported by studies proposing functional equivalence of spatial representations from touch and vision^28–30^. Furthermore, it was shown that blindfolded adults showed a tendency to visualize even non-visual stimuli^31–34^. However, previous research on spatial scaling in the haptic domain yielded inconclusive findings^8–10^ and does not allow to clearly identify specific spatial scaling strategies. Two of these studies did not separate the exploration process from placing the target in the referent space^8,9^. Moreover, two of these studies did not assess participants’ response times^9,10^. Hence, in the present study, we addressed these methodological requirements with the goal to analyze participants’ applied strategies.

### The present study

In the current study, we aimed to identify strategies used for spatial scaling in the haptic domain using a novel, methodological approach that overcame the constraints of previous research in the field. First, we systematically manipulated the size difference between the map and the referent space, creating three different scaling factors. Second, we separated the phase of perceiving the map from scaling this information from memory. Third, we measured response times and errors in the localization task.

## Methods

The current experiment was conducted in accordance with the 1964 Helsinki declaration and its later amendments or comparable ethical standards and was approved by the ethics committee of the Institute of Psychology of The John Paul II Catholic University of Lublin, Poland. Written informed consent was obtained from all participants included in the study prior to data collection.

### Participants

Thirty-one adults aged 20–24 years (*M*_age_ = 21.68 years, *SD* = 1.11; five males) participated in the current experiment. This number of participants is larger as the minimum sample size of *N* = 28, that is required in order to detect a within-participant effect of scaling factor in a repeated measure analysis of variance (ANOVA) as computed with a power analysis using G-Power 3.1^35^. This power analysis was based on a moderate effect size of *f* = 0.25 (based on findings from^5^), significance levels of *p* < 0.05, and a power of 0.80. Therefore, it seems that our analyses are sufficiently powered in order to detect effects of scaling factors. All participants of the current study were right-handed psychology students who took part in the study for course credit.

### Materials and design

**Figure 1:**
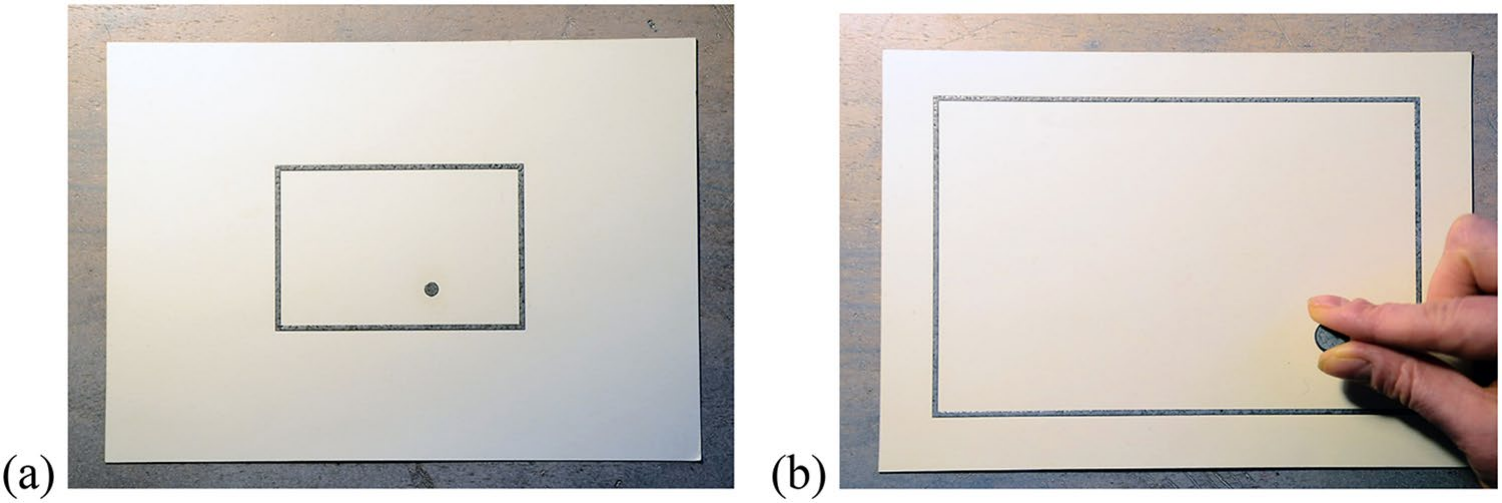
Example of a map for the scaling factor of 1:2 (**a**) and for giving the response in the reference space (**b**). The silver-grey elements of the boards are embossed.

We used 22 boards (148.5 mm high × 210.0 mm wide) containing embossed graphics which were made of cardboard (for analogous stimuli, see^8–10^) and a 10-mm large disc that participants used to respond (see Fig. 1). One of these boards represented the referent space. This referent space was indicated by a convex rectangular shape (110.0 mm high × 170.0 mm wide) centered on the board. Additionally, there were 21 boards representing the maps. Analogous to the referent space, their size was indicated by a convex rectangular shape centered on the board. By contrast to the referent space, these maps included a convex spherical target at one of seven different locations (see Table 1).

**Table 1:** Diameter of targets (in mm) and target locations (in mm) for different scaling factors (SFs).

Diameter of targets	SF 1:4		SF 1:2		SF 1:1	
	2.5		5		10	
Target location	X-coordinate	Y-coordinate	X-coordinate	Y-coordinate	X-coordinate	Y-coordinate
1	4.375	21.25	8.75	42.5	17.5	85
2	10	6.25	20	12.5	40	25
3	15.625	21.25	31.25	42.5	62.5	85
4	21.25	13.75	42.5	27.5	85	55
5	26.875	6.25	53.75	12.5	107.5	25
6	32.5	21.25	65	42.5	130	85
7	38.125	6.25	76.25	12.5	152.5	25

Sizes of the maps corresponded to three different scaling factors (1:4, 1:2, 1:1). Therefore, maps ranged from 27.5 mm × 42.5 mm (equivalent to scaling factor: 1:4), to 55.0 mm × 85.0 mm (scaling factor: 1:2), and to 110.0 mm × 170.0 mm (scaling factor: 1:1). The diameter of the targets ranged accordingly from 2.5 to 10 mm (see Table 1). The same seven target locations were used for the three scaling factors, amounting to a total of 21 maps. Three additional boards with empty spaces were used in practice trials prior to the test trials (a convex rectangular shape in three sizes: 27.5 mm × 42.5 mm, 55.0 mm × 85.0 mm, 110.0 mm × 170.0 mm).

### Procedure and coding

Participants were tested individually in a single session lasting approximately 30 min. During the experiment, they sat at a table and boards were placed subsequently on the table in front of the participant. The experimenter sat opposite to the participant at the other end of the table. Participants were blindfolded prior to the start of the study and got acquainted with the boards by touch. The spatial scaling task began with practice trials in which the experimenter explained the task. In these practice trials, participants were presented with empty boards in three sizes and were told that in subsequent trials, the space would contain a target represented by a dot. Next, participants were presented with 21 test trials in a random order. Each trial consisted of three stages (for a similar procedure, see e.g.^23^): (1) perceiving and learning the map and its target, (2) remembering and imagining the map at a given scale (ranging from 1:4 to 1:1), and (3) mapping the location of the target on the empty referent space from memory. The time of exploring the map in the first phase was fixed: the experimenter placed the board on the table and measured 20 s from the moment the participant began to touch the board. This amount of time was chosen based on findings of previous studies in the haptic domain^8,10,36^. In the first set of these studies^8,36^, the learning time was either fixed to 30 s or in case of unrestricted learning time amounted to approximately 30 s. In another previous study focusing on spatial scaling in the haptic domain^10^, the unrestricted learning time for simpler maps than used in the current study (targets varied on the horizontal dimension only) was less than 20 s on average. In the second phase, corresponding to the scaling factor (e.g., 1:2), participants were asked to imagine the maps on a given scale (e.g., to double the imagined map). The experimenter measured participants’ response times of this imagery task (in s, using a stopwatch) from the moment the participant was instructed until the participant signaled that he or she had imagined the map by saying "ready". This response time was taken as an indicator of the duration of the instructed spatial scaling process. Therefore, this stage was very similar to tasks used in mental imagery research^37^. During the third phase, participants were asked to locate the disk from memory on the referent space, putting it at the same location as the target presented in the map. At the end of this mapping, the accuracy of each response was assessed (by measuring values of the x- and y-coordinates in mm by the experimenter, using a ruler). Absolute errors that reflect the distance between a participant’s answer and the correct target location were calculated based on the x- and y-coordinates using the Euclidean distance formula.

## Results

### Response times

The analysis of variance (ANOVA) with participants' response times as dependent variable and scaling factor (1:1 vs. 1:2 vs. 1:4) as a within-participant variable yielded a significant effect of scaling factor, *F*(2, 60) = 15.66, *p* < 0.001, *η*_*p*_^*2*^ = 0.34, which was best described by a linear function, *F*(1, 30) = 26.68, *p* < 0.001, *η*_*p*_^*2*^ = 0.47. Participants showed higher response times with increasing scaling factors (for descriptive statistics, see Table 2).

**Table 2:** Mean absolute errors (in mm), and response times (in s) as a function of scaling factor (1:4, 1:2, 1:1). Standard deviations are presented in parentheses.

	Scaling Factor		
	1:4	1:2	1:1
Response times	5.98 (4.33)	5.59 (4.25)	3.62 (2.91)
Absolute errors	28.91 (15.66)	25.10 (13.10)	19.64 (12.92)

### Absolute errors

We computed a similar ANOVA with participants' absolute errors as dependent variable. This analysis showed a significant effect of scaling factor, *F*(2, 60) = 8.35, *p* < 0.001, *η*_*P*_^*2*^ = 0.22, which was best described by a linear function between scaling factor and absolute errors, *F*(1, 30) = 12.69, *p* = 0.001, *η*_*P*_^*2*^ = 0.30. Participants responses were more error-prone with increasing scaling factors (see Table 2).

### Signed errors

To see whether participants produced systematic directional errors^38,39^, we analyzed horizontal signed errors^40^. We calculated these errors by subtracting the x-coordinate of the respective target location from the x-coordinate of each participant’s answer (in mm). Negative signed errors indicate answers located too far to the left on the referent space; positive signed errors indicate answers located too far to the right on the referent space.

**Figure 2:**
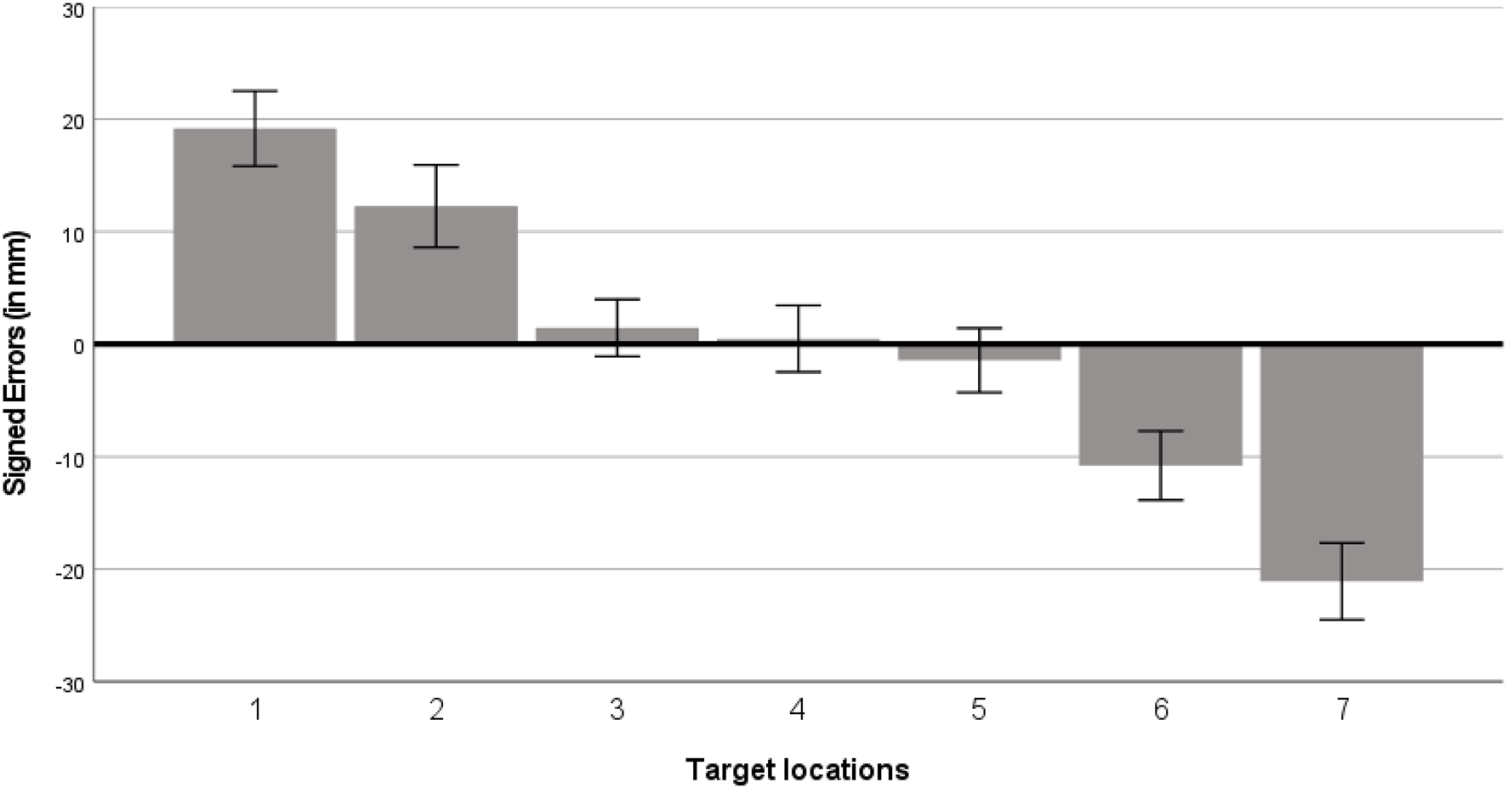
Participants’ horizontal signed errors (in mm) for different target locations, collapsed across the scaling factors. Target locations on the X-coordinate accorded to: 1 = 17.5 mm, 2 = 40 mm, 3 = 62.5 mm, 4 = 85 mm, 5 = 107.5 mm, 6 = 130 mm, 7 = 152.5 mm. Error bars represent ± 1 standard error. Positive signed errors indicate answers located too far to the right; negative signed errors indicate answers located too far to the left on the referent space.

We computed an ANOVA with signed errors as dependent variable and horizontal target locations (7, see Table 1) and scaling factor (1:1 vs. 1:2 vs. 1:4) as within-participant variables. This ANOVA showed a significant effect of target locations, *F*(3.33, 99.84) = 19.35, *p* < 0.001, *η*_*p*_^*2*^ = 0.39, that was best explained by the linear function, *F*(1, 30) = 40.56, *p* < 0.001, *η*_*p*_^*2*^ = 0.58. Participants produced larger directional errors for the peripheral locations as compared to the central ones (see Fig. 2). The analysis did not yield a significant effect of scaling factor, *F*(1.62, 48.64) = 0.25, *p* = 0.736, *η*_*p*_^*2*^ = 0.01, nor a significant interaction between target locations and scaling factor, *F*(7.08, 212.41) = 1.04, *p* = 0.402, *η*_*p*_^*2*^ = 0.03. Therefore, it seems that participants gravitated towards the midpoint of the space.

## Discussion

The current study investigated adults’ spatial scaling from memory in the haptic domain. Importantly, we tested spatial scaling abilities in this domain while addressing methodological constraints of previous research^8^. Findings of the present study suggest that participants used mental transformation strategies in order to solve the spatial scaling task. In analogy to mental imagery research^37^, mental transformation strategies are typically assumed when both absolute errors and response times increase linearly with higher scaling factors. Other spatial scaling strategies were associated with different patterns of findings^12,13^ that were not reflected in our findings.

More concrete, it was found that response times increased with higher scaling factors, in analogy to findings of mental imagery research^15,16^, and in line with previous related research in the tactile domain^23,28^. Results of absolute errors mirrored the ones of response times, in that absolute errors increased with higher scaling factors. This pattern of results extends prior studies that did not reveal clear signs that blindfolded participants used mental transformation strategies^8–10^. In contrast to these previous studies, in the current study, participants were asked to scale distances from memory whereas in previous studies, the map was available during the entire spatial scaling task^8,9^. Performing the task from memory may have increased participants' tendency to use an allocentric reference frame (i.e., visualize the map holistically), based on research showing that a delay between target perception and response resulted in a more allocentric performance pattern (for a review, see^41^). The assumption in which mental transformations are linked to an allocentric reference frame may be supported by studies suggesting that people use holistic mental representations when visualizing spatial haptic information^10^. Other studies demonstrated that sighted, blindfolded adults (but not congenitally blind adults) used allocentric spatial representations when perceiving stimuli haptically (in other words, when the tactual-kinesthetic system is involved)^42^. However, as we did not directly measure which reference frame adults have used in the current study, future research may investigate this topic systematically.

Crucially, for the first time, the current study identifies mental transformation strategies in the haptic domain and supports results from studies in the visual domain^5,13^. Given that similar strategies can be found in adults’ spatial scaling across different modalities, our findings support the notion of functional equivalence of cognitive map formation and processing from touch and vision^28–30^. Additionally, we observed directional bias in adults’ localization errors as indicated by adults’ signed errors. The linear pattern of results indicates that blindfolded, sighted participants tended to gravitate towards the middle of the perceptual space, and thus, represented the spatial layout as a single entity. Similar findings were shown in the previous studies on spatial scaling in the haptic domain^8,10^.

### Limitations and suggestions for future research

The current study has several limitations. First, when taking the debate on the nature of mental imagery into account (^43–46^ vs.^47–49^; see also^50^), one may argue that participants of the current study were provoked to use a mental visualization (i.e., using a depictive representation), and consequently adopted a mental transformation strategy to solve the imagery task^15,16^. However, it needs to be noted that participants were not explicitly asked for "creating a mental picture of the map" nor to "mentally zoom spatial information". As shown in a study on estimating object sizes in the visual and tactile domain^23^, the size can be estimated by the participants verbally (e.g., in centimetres). Thus, it was also possible that in the current study, participants would have used a verbal relative distance strategy by estimating the distances between the target and map boundaries, and increasing this distance proportionally when being asked to scale this information.

Second, previous related research has used isochrony measures based on the concept of functional equivalence between actual and mental movements^51^, which further supports the claim of applying analog mental transformations strategies. However, in the current study, we did not measure response times of participants’ actual movements (i.e., when they would physically transform the spatial layouts). Future research may consider using isochrony in order to strengthen the claim of using mental transformation strategies.

In our view, the present research could be further developed in at least two directions. In the current study, targets on the maps varied on two dimensions (horizontal and vertical). When comparing the absolute errors in adults’ spatial scaling performance in the tactile domain (as reported in^9^) with the present findings, it can be concluded that a 2-dimensional condition is more demanding than a 1-dimensional condition (i.e., when targets vary on the horizontal dimension only). Similar results have been found in studies on children’s spatial scaling and indicate a higher complexity of 2-dimensional as opposed to 1-dimensional target distributions^1,7^. With respect to dimensionality, it is possible that participants may use different strategies for maps characterized by 1- or 2-dimensional target distributions and future research may identify whether participants apply different strategies when target distributions differ.

Furthermore, it seems possible that participants with congenital blindness who are able to effectively use tactile maps^28,52–54^ but cannot visualize maps would implement different scaling strategies when performing the present task^32–34^. Blind individuals are more familiar with tactile maps than sighted people, and this expertise seems vital in their daily life. Hence, identifying spatial scaling strategies used by blind participants with varying experience with tactile maps may further elucidate this issue.

## Conclusions

Overall, the present study indicated mental transformation strategies by blindfolded adults for the first time and thus, qualified previous studies in the research field on spatial scaling in the haptic domain^8–10^. In addition to this outcome, the present study provides a novel methodological approach in order to investigate spatial scaling in the haptic domain, enabling to address methodological constraints of previous research.

## Data Availability

The dataset used and analysed during the current study is available from the corresponding author on reasonable request.
